# Living the 14/14 Schedule: Qualitative Analysis of the Challenges and Coping Strategies among Families of Offshore Wind Workers

**DOI:** 10.3390/ijerph16020241

**Published:** 2019-01-16

**Authors:** Janika Mette, Swantje Robelski, Maria Kirchhöfer, Volker Harth, Stefanie Mache

**Affiliations:** Institute for Occupational and Maritime Medicine, University Medical Centre Hamburg-Eppendorf, 20459 Hamburg, Germany; s.robelski@uke.de (S.R.); maria.kirchhoefer@googlemail.com (M.K.); harth@uke.de (V.H.); s.mache@uke.de (S.M.)

**Keywords:** offshore wind industry, reconciliation of offshore work and family life/partnership, coping strategies, psychosocial adaptation, qualitative analysis

## Abstract

Offshore wind workers in Germany usually spend 14 days offshore, alternating with 14 days of spare time at home. The offshore lifestyle may considerably affect offshore workers’ partners and families. However, there is a lack of evidence regarding the psychosocial adaptation among offshore wind couples living the 14/14 schedule. The present study intended to offer a contemporary view on the topic from the perspective of the women of offshore workers. Our aim was (1) to examine the perceived features of living the 14/14 schedule, (2) explore women’s coping strategies, and (3) investigate their views on the reconciliation of offshore work and partnership/family life. The women reported differentiated views on the benefits and costs associated with their living situation, and stated various coping strategies that facilitated psychosocial adaptation. Despite some burdens, overall, most of the women seemed to have adapted relatively favourably to their lifestyle. This was particularly eased by recent sociological and technological advances, e.g., improved communication technologies.

## 1. Introduction

In Germany, coastal areas of the Northern and Baltic Sea offer possibilities for the construction of offshore wind parks. The growth of the German offshore wind industry as part of the green energy revolution is shown by recent figures: in the first six months of 2018, 62 new wind energy convectors were taken into operation [1]. All in all, there were 20 German offshore wind parks by the end of June 2018, generating about 5.300 megawatt (MW) [2]. This development goes along with an increase in employment figures in the branch. Recent estimates indicate that approximately 27,200 workers, most of them men, are currently employed in the German offshore wind industry along the value chain [3].

Offshore wind jobs are associated with many hardships, as well as physical and psychological demands for their employees [4,5,6,7]. A key element of working in the German offshore wind branch is the 14/14 work schedule that is applied: offshore wind workers usually spend 14 days offshore, where they work in 12-h-shifts and live on platforms, ships, or neighboring islands. These periods of leave alternate with 14 days of spare time the workers spend at home. From a psychosocial viewpoint, this schedule implies that the workers are confronted with a phase of absolute separation from their families and social environment, followed by an intensive free time period spent together. The 14/14 work schedule is unique and qualitatively different from other work patterns, e.g., work schedules of shift workers or employees with on-call-work. While the latter jobs still allow off-duty time at home each day, offshore workers spend entire weeks away [8]. The 14/14 schedule of workers in the offshore wind industry also deviates considerably from the schedules of other commute workers: the periods of absence of offshore workers are longer than those of commuters coming home for the weekend, but shorter than those of, e.g., seafarers in the maritime sector spending months away from home.

It has recently been noted that the recurrent absences from home represent a job demand for many offshore wind workers, leading to difficulties in reconciling offshore work and family life [5,6,9]. However, the specific lifestyle of the offshore personnel does not only influence the workers themselves, but may also affect their partners and families at home who must deal with the repeated partings, reunions, and phases of intermittent absence. For example, female partners of offshore workers may find it challenging to manage all domestic and parenting tasks alone, to deal with the disruption to shared family activities, and to establish routines in their daily life. Likewise, there could be certain features of the 14/14 schedule that may potentially enhance family life, e.g., the chance to continuously spend intensive time together on a regular basis.

The notion of women experiencing difficulties in dealing with the intermittent absences of their partners is supported by studies carried out in branches with extended periods away from home, e.g., the offshore oil and gas branch and mining industries [8,10,11,12]. Research from the offshore oil and gas sector has indicated that partners staying at home experience various demands on their own [8], being condensed within the statement of “living two lives”. Moreover, early research on the intermittent presence and absence of offshore oil and gas workers has differentiated between three perceived phases for the couples: her single life at home, his offshore life, and their common life together [11]. Specific demands for the women, as revealed in previous studies, consisted of experienced negative emotions, as well as difficulties related to role allocation and family structures [8,13].

Similar to offshore branches, mining industries are also characterized by long distance commuting and specific rosters. They are often referred to as fly-in fly-out (FIFO) branches. The presence of the partner at home is also rather frequent and extended in the FIFO branches in comparison to other industries with prolonged absences. It has been proposed that the living situation of FIFO couples may be described as a cycle with the stages reunion, time commonly spend together, parting, and time alone [12]. In this cycle, every stage was associated with certain demands, adjustments, and negotiations to be made by the couples, and with a range of perceived emotions attached to the transitions.

Although the offshore lifestyle was found to be related to specific demands, an early study among so-called offshore wives did not find differences between the mental and physical health of offshore and onshore wives [14]. Likewise, recent research results suggest a healthy functioning range for psychological wellbeing, relationship satisfaction, and perceptions of family function in FIFO workers and their partners [15]. Further research on the health of FIFO families showed a generally high level of family cohesion, healthy flexibility, and a general contentment with regard to family satisfaction and communication [10].

The ability to deal with the challenges encountered by partners of commute workers may partly depend on the coping strategies being applied. According to the transactional approach of Folkman and Lazarus [16,17], coping can be defined as the cognitive and behavioral efforts made to master, tolerate, or reduce external and internal demands, as well as conflicts among them. Coping efforts may either concern the management of the stress-inducing problem (problem-focused coping) or the regulation of emotions or distresses (emotion-focused coping) related to the situation encountered [16,17]. Research among FIFO families has, for example, indicated the existence of different coping strategies of the couples to deal with the difficulties they have encountered. As described in an interview study, such strategies encompassed maintaining open communication, parting on good terms and without major arguments or conflicts, and generally maintaining a positive attitude towards the situation [12].

Despite existing research studies in FIFO and offshore oil and gas industries, there is a lack of evidence regarding the situation of offshore wind couples living the specific 14/14 schedule. Findings for couples in the oil and gas and FIFO branches are not directly applicable to the situation of couples in the offshore wind industry, since the work schedules in the branches can differ considerably, and employees in the afore mentioned branches usually do not follow the 14/14 work pattern as applied in the German offshore wind branch. Moreover, there are further differences between the branches, e.g., regarding specific regulations, work areas, and work tasks [4,6,18].

In addition, much of the existing research on offshore couples dealing with intermittent absences was conducted in the 80’s, 90’s, and early 00’s, indicating a need for an updated view. Within the last decades, profound organizational, technological, and sociological changes have taken place which pertain to offshore industries [8]. For example, the extension of means of communication provides couples with further possibilities to keep in regular contact. Changes in society have also occurred in terms of women’s qualifications and employment rates, making it more likely that women of offshore workers are engaged in paid work. Finally, as a corollary of social changes, women’s role models and aspirations have evolved: nowadays, women tend to have stronger expectations as to their husband’s involvement in childcare and housekeeping [8]. Such recent changes could have either beneficial or detrimental impacts on the living situation of couples dealing with intermittent absences, which has not yet been empirically explored.

Since a further increase in the offshore wind workforce is to be expected, more couples and families will have to deal with the particularities of living the 14/14 schedule. Therefore, it is crucial to conduct up-to-date research and generate new knowledge on the specific features of this lifestyle. This study intended to offer a contemporary view on the psychosocial adaptation of offshore wind families against the background of the modern society and working world.

The aim of our study was to examine how women of male offshore wind workers perceive the specific features of living the 14/14 schedule. In adherence to the approach by Parkes and colleagues [8], we focused on the different phases of women’s daily life, including aspects of being home alone and time spent together as a couple. Moreover, our purpose was to investigate the coping strategies of the women and the couples to deal with the specific living situation. Furthermore, we aimed to explore the women’s perceptions of the reconciliation of offshore work and partnership/family life.

We proposed the following research questions:

1. What are the advantages and disadvantages of living the 14/14 schedule as perceived by the women of offshore wind workers, particularly regarding the following phases:
  (1) life without offshore partner;
  (2) life together as a couple/family;
  (3) transition phases (reunion/parting)?
2. What coping strategies are employed by the women and couples to deal with the specific features of living the 14/14 schedule?
3. How do the women judge the reconciliation of offshore work and partnership/family life, including aspects of organizational support?

## 2. Materials and Methods

### 2.1. Study Design and Participants

We conducted 14 semi-structured telephone interviews with female partners of German offshore wind workers from January to March 2017. The interviews were carried out by two female psychologists working as researchers in occupational health psychology at the time of the study. We applied purposeful sampling and recruited interviewees of different ages, with and without children, and with offshore partners working in different companies. Participants were eligible if they were female, fluent in the German language, and at least 18 years old. Moreover, the females’ partners had to have worked for at least six months in the offshore wind industry, and had to have experiences with the 14/14 work schedule. To recruit suitable participants, we sent invitation mails, emails, and leaflets to German offshore wind workers from different companies who had already participated in a prior interview study conducted at the research institute [5,6]. Moreover, we posted the study information on online platforms and forums for German offshore wind workers. We asked the workers to inform their female partners, who then contacted us directly via mail or telephone. Study participation was voluntary. Prior to the interviews, participants were asked to sign a declaration of informed consent. All participants were in a position to understand and consent to the study requirements, and provided written informed consent. The interviews were conducted until no new themes were identified, i.e., data saturation was reached. They were conducted in German and were tape recorded. Interview length was from 28 to 54 min. Participants were able to terminate the interviews at any time. No non-participants were present during the interviews. No repeat interviews were carried out. Field notes were made immediately after each interview.

### 2.2. Interview Guideline

A semi-structured interview guideline was developed within the framework of the empirical and theoretical background. The interview topic list is depicted in Table 1. A pre-test interview was performed in order to receive feedback from research colleagues and improve the interview guideline.

### 2.3. Analysis

All audio recordings were transcribed verbatim. The transcripts of the interviews were anonymized and analyzed in a deductive-inductive process according to Mayring’s qualitative content analysis [19] by means of the software MAXQDA Analytics Pro, version 12 (VERBI Software GmbH, Berlin, Germany) [20]. An iterative process was applied in which the authors identified and refined codes, categories, and sub-categories. The coding was mutually checked for accuracy and was thoroughly discussed until consensus regarding the final coding system was reached. The final coding system was summarized in a separate document in which the material was further reduced and compacted. During the course of analysis, reflexivity and transparency regarding the potential influence of the researchers’ objectives and preconceptions on the results and interpretations were encouraged. Transcripts and results were not returned to the interviewees. All quotes that were used for publication purposes were translated into English.

## 3. Results

### 3.1. Sample Characteristics

As illustrated in Table 2 and Table 3, seven women were aged between 31 and 40 years old; 10 women were married; seven women reported to work full-time, two worked part-time, and five were currently on maternity or parental leave; and eight women were mothers of children living in the household at the time of the study. The partners of 10 women had worked offshore from the beginning of their partnership. Furthermore, 12 offshore partners were currently working in a regular 14/14 work schedule, and two partners were working in a different schedule at the time of the interview but did have experience with the 14/14 schedule. Additionally, seven partners had at least three years of offshore work experience.

### 3.2. Single Life without the Offshore Partner

#### 3.2.1. Advantages

As a major advantage of living the single life, many women found themselves to be more independent and self-reliant in their scheduling of activities and appointments. This was also reported regarding duties and routines within the household and parenting:

“*Very simple things, like, for example, when to make purchases, what to buy, when to clean certain things (…).*”

*(Interviewee #8)*

A further advantage of the time spent alone was that the women pursued their own interests to a greater extent and were able to become engaged in several leisure activities:

“*Advantages, perhaps, that one deals with being alone and tries to find strategies to better cope with it. You do not rely on the fact that there is always someone there, but you put more thoughts in your own leisure activities.*”

*(Interviewee #4)*

Meeting friends and family, doing sports, and following diverse hobbies were stressed by the women as important leisure activities in this phase.

#### 3.2.2. Disadvantages

An important disadvantage of living the single life was reported to consist of the management and organization of daily life without the partner’s general support. Spare time was described to be affected by the partner’s absence in such way that the women perceived being forced to spend big parts of their social life alone:

“*A disadvantage is, of course, that you can only maintain your social contacts alone. If there are invitations or birthday parties, or if you just want to spend a nice evening with friends, this doesn’t work.*”

*(Interviewee #13)*

During their time alone, there were moments in which the women especially missed their partners. These often concerned moments that both partners usually shared, in particular regarding time during the evenings or weekends. Difficult situations such as sickness, problems at work, or issues with childcare were also mentioned. Moreover, the partner’s being missing on special occasions, e.g., weddings or special moments in the children’s development, was emphasized. Some interviewees indicated emotional changes, e.g., feeling lonely or more tense than usual:

“*I realize that I’m not feeling well when he’s not there. Sometimes I do not sleep for nights because I cannot stand it.*”

*(Interviewee #5)*

Another disadvantage consisted of perceived insecurities related to child care and parenting. Some interviewees described that they considered themselves as single mothers when their partners were offshore:

“*Well, more or less, I am a single parent for two weeks.*”

*(Interviewee #9)*

The perceived unpredictability, e.g., regarding the exact time of the partner’s arrival back home, was also seen as disadvantageous. Moreover, the awareness of constraints in reachability when the partner was offshore—especially in cases of emergency—was highlighted as burdening.

Many women spoke about changes in their work routines when their partners were away. They described often working overtime during this phase in order to be able to work less or leave on time when the partner was at home:

“*Of course, when my husband is not there, I work more. When he’s at home, I scale that back a bit, so that we have more time for each other.*”

*(Interviewee #9)*

Especially women with children found themselves to be less flexible and more constrained in their work times when they were alone, e.g., due to their children’s schedules. Some mothers also described that their children’s ill-health seemed to be connected to the absence of their father:

“*It also happens that the children get sick if he is not there for a long time.*”

*(Interviewee #6)*

#### 3.2.3. Communication and Contact Styles

Staying in contact with the partner during his offshore assignments was reported to be crucial for the women. Most of them described being able to contact their partners on a daily basis. The scheduling of contact was stated to depend on the shifts and workloads of the partner:

“*During his shift, I can write him and if he reads it in between, he can also answer me. But we rather talk on the phone during his free shifts.*”

*(Interviewee #12)*

Certain difficulties and restrictions to make contact were also described by some women; however, contact that could be initiated by both partners was reported to be the rule. The usage of several media (phone, messenger services, video telephony) was mentioned by the majority of the women. In particular, improved communication technologies were stated to enable more intensive contact and direct exchange. All in all, the majority of the women stated being satisfied with both the frequency and regularity of contact. Reasons for dissatisfaction were related to the time of contact and the general notion that technology-mediated contacts were not comparable to personal contacts.

### 3.3. Life as a Couple

#### 3.3.1. Advantages

Advantages of the 14/14 work schedule were often attributed to the time spent together as a couple during the partners’ free turn. This time was stated to provide the couple with much time for joint activities and family life. The family life was perceived as being even more intensive due to the previous phase of separation, providing the couple with an opportunity to miss and look forward to seeing each other again. This was indicated to contribute to the liveliness of the relationship:

“*It’s also good for our relationship, it’s good to have such a short break (...). That brings in a certain freshness.*”

*(Interviewee #6)*

The presence of the offshore partner at home was reported to allow for a higher quality of communication through face-to-face talks. A further advantage of the time spent together was that the partner could be involved in the child care and housekeeping. Family fathers were also able to follow their children’s development more closely during their free time at home.

More generally, some women described time-wise benefits of the daily living together, such as a greater flexibility for the couple in terms of planning short vacations or having breakfast together during the week. Financial benefits of the offshore job (e.g., in the form of good salaries) that contributed to family life were also mentioned.

#### 3.3.2. Disadvantages

A main disadvantage of the daily living together in the 14/14 schedule concerned the lack of habitualness and missing daily routine which could not be established in the course of two weeks:

“*We are all habitual people and habits can be very difficult at 2 weeks/2 weeks, I think.*”

*(Interviewee #11)*

A few interviewees described feeling an increased need to talk to their partners and to plan appointments when they were home. Some women even described a perceived pressure to get all everyday things—for which they normally had four weeks of time—done during the two weeks together:

“*You always have the feeling that you must put everything into these two weeks, because afterwards, your time together is over. Need for action, discussion needs… what you just can’t always hold on the phone.*”

*(Interviewee #9)*

In terms of their work, many women described their own job as meaningful to them, regardless of the presence or absence of their partners. However, some women found it harder to go to work with their partners being at home and preferred staying at home with them, e.g., because they experienced feelings of guilt for leaving the partner alone.

In addition, some women reported that the situation was especially disadvantageous for their partners: their free turns could be rather unsatisfactory, since the time they were able to spend with their families and friends was restricted due to other people’s normal work routines:

“*It was not satisfactory for him either. He was not socializing as much as he had wished. The days are long when all people around you work full time.*”

*(Interviewee #11)*

Other interviewees explained that their partners struggled to find a balance in terms of the time spent with the family and with friends outside home. This was due to the fact that the workers were solicited a lot during their onshore turns, in particular on the weekends:

“*The time on the* *two weekends becomes very, very scarce. When there should be time for the partnership, but also for family, friends, and your own interests.*”

*(Interviewee #4)*

Several women described that their partners absolved work tasks and were contacted for professional purposes during their free turns onshore. Talking about offshore work and being contacted by colleagues were associated with greater difficulties for the partners to mentally detach and recover from work. Therefore, many interviewees disapproved of this behavior.

#### 3.3.3. Conflicts and Compromises

When asked about conflicts and compromises due to the specific living situation, about half of the women stated not noticing any specific conflicts. The non-existence of conflicts was attributed to the couple’s mutual understanding and awareness of their limited time together:

“*Because we are separated again and again, you appreciate it (the time spent together) very much. And that makes us both feel that we are not arguing so fast and so much.*”

*(Interviewee #10)*

The other women reported that minor conflicts or discussions attributable to their specific living situation sometimes occurred. Conflicts, for example, emerged when the partner refused to get involved in housekeeping, or when he dedicated too much time to his work during his free turn. Further discussions were described to relate to planning difficulties of the couple due to the partners’ offshore work. Moreover, minor discussions between the offshore partner and the children were reported to occur. In addition, it was stated that ‘offshore couples’ sometimes had to deal with a lack of understanding from their friends, who did not comprehend the amount of time the couple needed for themselves.

The majority of the interviewees thought that they had to make more compromises compared to couples living a ‘normal life’. They expressed that more agreements and consultations were necessary to suitably plan living together. Compromises were, for example, described in terms of the parenting, since the children had to live without their father for a while. Moreover, planning difficulties were a central concern, since all appointments had to be made in accordance with the partner’s offshore schedule:

“*We have to direct our everyday life according to these offshore trips. He never knows when the trips will be —they are not set at the beginning of the year—so we just cannot plan at all.*”

*(Interviewee #5)*

### 3.4. Transition Phase

#### 3.4.1. Reunion with the Partner

The women described varying feelings, e.g., increasing anticipation and excitement, upon their partners’ arrival back home. Typical behavior patterns were tidying up the house and avoiding other appointments:

“*Then I just run from A to B and check that everything is neat (…). That the food is ready and that no more laundry is lying around. That there are no disruptive factors in order for us to simply enjoy this moment together.*”

*(Interviewee #5)*

Only a few interviewees stated that they did not perceive a certain transition phase when their partners arrived back home. The majority declared that they needed a familiarization phase in which they had to adapt to their partner and the two adult household again:

“*At the beginning, you often need some time to get close again, because you have not seen the other person for so long.*”

*(Interviewee #7)*

The transition phase was generally described to last between one and four days. Women described that the arrival of the partner could upset the whole household, and that the habits and routines of the women at home were suddenly turned around:

“*You develop different habits—your own habits—when the partner is not there. And as soon as he comes back, it’s all jumbled up.*”

*(Interviewee #8)*

In households with children, it was pronounced that the children behaved more actively and turned up during the father’s arrival, demanding more attention than usual:

“*When he comes back, the children are usually there, and then the alarm goes from 0 to 100 in the booth.*”

*(Interviewee #13)*

#### 3.4.2. Needs and Expectations upon the Partners’ Arrival

Needs and expectations of the women regarding the time as a couple consisted of spending as much time together as possible and following social activities. Some women particularly highlighted their expectation that the partner should get involved in housekeeping and other duties at home:

“*I indeed expect that he will also take care of the household and of the things that happened while he was not there.*”

*(Interviewee #13)*

In contrast, expectations of the offshore partners stated by the women included that the women should await them at home upon their arrival and that the couple should share a good meal together on the first evening. A relevant need of the partner consisted of physical closeness to the women.

Some interviewees believed that their needs and expectations corresponded well with those of their partners; for example, when both partners wished for physical closeness, calmness, and time spent together. In contrast, other women perceived discrepancies, which were especially related to the women’s “work situation” versus the partners’ “free time situation”: while the women had to continue their daily work routine, the offshore partners found themselves to be in a holiday mood:

“*I get up at the same time in the morning, go to work, and come back in the evening. And then my partner took the time as a vacation, but I was still in the working cycle.*”

*(Interviewee #4)*

#### 3.4.3. Parting

The interviewees described that the time spent together as a couple usually passed rapidly. Some women described that during the last days before their partners’ departure, the workers started to mentally prepare themselves for their offshore assignments. The departure was termed as a difficult situation by some women, provoking feelings of sadness. Some women also reported that their children’s behavior changed during their father’s departure. For example, they could demonstrate their displeasure by crying or working themselves up.

**Figure 1:**
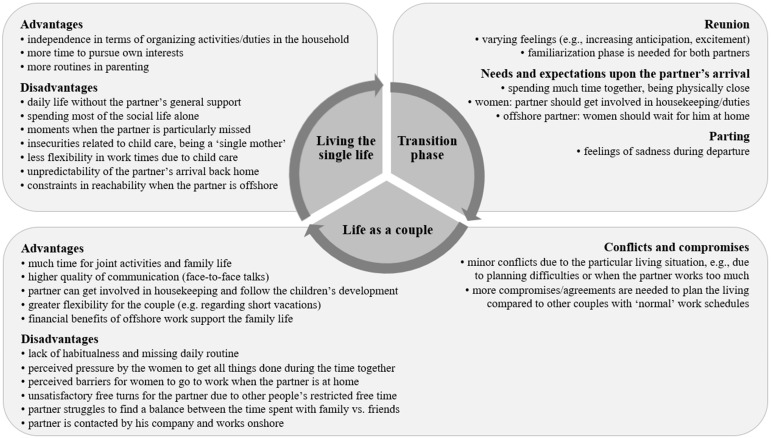
Specific features of living the 14/14 schedule related to the phases of daily life.

Figure 1 gives an overview of the specific features, advantages, and disadvantages of living the 14/14 schedule as related to the different phases of daily life.

### 3.5. Coping Strategies

#### 3.5.1. Strategies of the Women

When asked about strategies to cope with the absence of their partners, many women described that actively searching for the support of families and friends played an important role:

“*I think that you rely more on the social network around you. That you particularly promote your network. You simply intensify other social contacts, family, friends.*”

*(Interviewee #4)*

In general, pursuing an active lifestyle was described as a coping strategy by many women. This included, for example, doing sports or meeting friends. Further ways to cope with the situation were stated to consist of adapting oneself to the schedule, focusing on the time spent together as a couple, and staying in regular contact during periods of separation:

“*For me, this is already somewhat normal. And we talk on the phone in the evenings and write each other during the evenings when he has enough time.*”

*(Interviewee #12)*

Some interviewees stated that they coped with the situation by structuring their time in an organized manner, while others reported that they coped by keeping their expectations low regarding the time spent together. Some women reported that they did not apply any coping efforts.

When being asked about sustaining exchange with other women living in a similar situation, most interviewees responded that they had not made contact with other women of offshore workers, although some found such an exchange to be desirable. A few others, in contrast, described having irregular contact with other partners of offshore workers. These contacts were reported to be organized autonomously and without the offshore companies’ support.

#### 3.5.2. Strategies of the Couples

In terms of coping strategies applied by the couples, many women emphasized the meaning of communication and structure for dealing with the phases of separation:

“*This always means a lot of exchange with each other, and a lot of talking and communicating. Then it works. But those who do not have this ability will find it difficult.*”

*(Interviewee #9)*

The importance of adhering to fixed and regular contact times was stressed. Additionally, spending time as a family/couple when the partner was onshore was underlined, explaining that other obligations or appointments were avoided in order to create more family-time:

“*Certain rituals are that, when he’s at home, (…) the last weekend before he leaves, or at least 1 or 2 days, that you have these days completely to yourself. And then accept no appointments.*”

*(Interviewee #10)*

Mutual understanding and trust were also highlighted as important. Still, there were couples who did not apply any coping strategies or rituals to deal with the specific situation.

Table 4 summarizes the coping strategies applied by the women and couples.

### 3.6. Reconciliation of Offshore Work and Family Life/Partnership

#### 3.6.1. Opinions on Reconciliation

The women’s views on whether or not they considered offshore work and family life to be reconcilable differed. There were some interviewees who described offshore work as being sufficiently family-friendly, especially when the children were already older in age. The primary reason for the family-friendliness was that fathers were able to spend intensive periods of time at home:

“*Which father of a family can say that he is completely at home for 2 weeks, from morning to night?*”

*(Interviewee #10)*

In contrast, other women did not consider offshore work to be family-friendly due to the several named disadvantages implied by the 14/14 schedule. This was particularly pronounced by interviewees with smaller children, emphasizing that the partner would miss out on too much of their development:

“*I just think that the men miss too much. Especially when a child is born. In the first year, our son actually had only me, his dad was always a bit of a rival.*”

*(Interviewee #5)*

A dividedness regarding interviewees’ opinions was also apparent with regard to the reconciliation of offshore work and partnership. Some women believed that offshore work was partnership-friendly and that it helped in keeping the partnership alive. Living with the periodical absences of the partner was reported to be practicable without children:

“*Without a child, I’d say, it works. Then you can deal with it, even without noticing a negative impact on the relationship.*”

*(Interviewee #4)*

However, other interviewees stated that the 14/14 schedule imposed heavy demands on the relationship, and that they personally perceived the situation as burdensome:

“*The big disadvantage is that the private life suffers greatly, that one must cut back on the partnership because the contact is missing.*”

*(Interviewee #5)*

The appraisal of whether or not offshore work was partnership-/family-friendly was stated to depend on the couples’ expectations (e.g., the amount of time the couple wanted to spend together and the desired frequency of contact). Moreover, the amount of support from external sources (e.g., parents, friends) played a role in the women’s judgement:

“*If you do not have family support, then it is not necessarily family-friendly. So I think that the environment still plays a big role.*”

*(Interviewee #7)*

#### 3.6.2. Needs and Wishes for Improving Reconciliation

When asked about wishes for improving the reconciliation of offshore work and family life/partnership, some women stated that they did not have any specific wishes, or explained that they did not think that any measures for improvement could be taken due to the unchangeable 14/14 schedule. Others, however, described wishes regarding their partners’ work schedule (e.g., other days of arrival and departure, longer offshore or onshore stays). The wish for greater regularity and predictability of the offshore assignments was also expressed:

“*These are my concerns, reliability and predictability.*”

*(Interviewee #6)*

Furthermore, it was proposed that the workers should get more free time offshore in order to increase chances for communication with the families and to strengthen the workers’ recovery from work. A few women also wished to get to know their partners places of work to develop a better understanding of the work situation offshore.

#### 3.6.3. Support from Offshore Companies

The women mostly described that they did not know about specific offers provided by their partners’ companies to facilitate the reconciliation of offshore work and family life/partnership. However, they described single offers provided by the companies that they considered to be helpful, e.g., flights back home at short-notice in case of emergencies:

“*Of course, if there was a death in the family or something, definitely. Or now with the child’s birth, I could call him anytime and would try to get him off the platform.*”

*(Interviewee #10)*

Some women said that company events were organized for the whole family, and that parental leave for fathers was an option. Further offers consisted of the free use of a telephone and internet connection on the offshore platforms, allowing the couples to stay in contact:

“*That’s a good option, I think, that companies put a lot of emphasis on enabling the workers to have regular contact with their families at home.*”

*(Interviewee #10)*

## 4. Discussion

By conducting our interview study, we were able to gain important insights into the challenges, advantages, and disadvantages, as well as the psychosocial adaptation, associated with living the 14/14 schedule from the perspective of women of offshore wind workers.

We generally found the proposed differentiation between the three distinct social realities for offshore couples (her single life at home, a phase of transition, and the couple’s common life together), as suggested by Solheim [11], to be similarly described in our study. Moreover, our results seem to be in line with the FIFO cycle proposed by Gallegos [12] for both offshore employees and their partners. For example, the occurrence of mixed emotions during transition phases, as identified in previous research [12,15], was also prevalent for the women in our sample.

Overall, despite some burdens, the women in our sample seemed to have adapted relatively favorably to the challenges and demands of living the 14/14 schedule. Although minor difficulties and problems related to the partnership and family life were stated, most women seemed to be able to cope with the challenges associated with the 14/14 work schedule. When contrasting our findings with those of earlier research studies in the offshore oil and gas branch, we found previous research to illustrate a slightly more negative picture regarding the psychosocial adaptation of offshore families [21]. However, the situation seems to have improved over the last decades. For example, Parkes and colleagues [8] noted a positive trend, and our study further supports this development.

### 4.1. Single Life without the Offshore Partner

As regards the single life of the women, we found the main advantage to consist of the greater self-reliance and independence women perceived in their daily living. This is in line with previous results showing that women were able to enjoy their independence and freedom [21,22], and that they could benefit from their partners’ absences in developing greater personal confidence [8]. However, in general, we found that the women in our study reported more disadvantages than advantages of their single life. Negative aspects, such as perceptions of loneliness when the partner was away, have been similarly revealed in previous studies [8,12]. For example, in an interview study, two thirds of the spouses of offshore oil and gas workers reported loneliness to be a problem “sometimes” or “often” [8].

In contrast to earlier studies in which women reported experiencing social isolation due to their partners’ absences [8,13,22], our interviewees did not describe difficulties in fully participating in social life during this phase. This difference could be related to the fact that the women in our study did not seem to center their social lives strictly around their partners; in contrast, many of them stated that they actively engaged in social life when their partners were away. In earlier studies, women were found to deliberately restrict their social lives when their partners were away [8,22].

An important finding of our study relates to the use and impact of new ways of communication as a result of technological advances. We found the women in our study to positively highlight their chances for communication via the use of diverse social media. While earlier studies declared problems in communication due to the—back then—existing communication systems [13], today’s offshore women may draw on several communication systems which allow them to keep in contact with their partners. The importance of improved telecommunications in facilitating adjustment and maintaining family connectedness has also been noted for offshore and FIFO families [8,10,22]. One study, for example, found that women who could initiate calls to contact their offshore partners had less difficulty in adjusting to the absence compared to women who were unable to do so [8].

Our results indicate an intrinsic work motivation among the women in our sample: becoming engaged in work was identified to be important, helping them to fill the days when their partner was offshore. In contrast to an earlier study in which women’s employment tended to increase family strain [23], we did not find this to be the case in our study. This discrepancy should be interpreted in view of the specific sample: six out of 14 women did not have children in the household, thereby potentially increasing their chances to engage in paid work. Furthermore, the result may also be attributable to sociocultural changes that have occurred during the last decades: nowadays, it is more common and socially accepted for women to build their own careers. The trend of increased employment rates among women is also reflected in our sample, in which all women were employed (despite five women currently being on parental or maternity leave). In contrast, in earlier samples of offshore wives, only one third [23] and two thirds of the women [8] respectively, were engaged in paid work. Similarly, only two women in our study were in part-time employment, whereas more than half of the women in the study of Parkes and colleagues [8] worked part-time, which was found to be influenced by the demands of childcare. The fact that most women in our study worked full-time may also be related to currently increasing options for child care, e.g., provided by day care centers and kindergartens.

### 4.2. Life as a Couple

With respect to life as a couple, the women in our study had differentiated views on the costs and benefits of the 14/14 schedule for their living situation. A major advantage related to the 14/14 schedule was seen in the workers’ rest periods onshore, allowing an increased duration of presence at home and favoring family life. This advantage was also noted in earlier studies [8,12,13,21].

As previously identified [11,12,24], we found that both partners initially needed a familiarization phase to readjust to having another adult in the household. Similar to our results, it was previously found that reunions and partings are the most difficult times emotionally for couples and families [10,12]. Moreover, in accordance with previous findings [12], women in our study reported that their routines could become less structured when their partners returned home.

In terms of the children’s behavior, some women described their children’s conduct as varying and depending on the phase of absence, presence, or transition. Gallegos [12] has similarly described that the behavior of children of FIFO workers could become clingy, and that it could take some time for the children to feel comfortable upon their father’s return.

Notably, there were only a few conflicts described as having occurred during the time spent together, although conflicts seemed to be a stressor for couples in previous studies [21,22,23], especially during the first days spent together [21]. In contrast to previous research highlighting women’s increased responsibilities as a potential source of conflict [13,15], conflicts described by our sample seemed to emerge from the partners’ behaviors at home, e.g., their reluctance to get involved in housekeeping. In earlier times, housework did not represent a source of conflict for offshore oil and gas couples [21]; the major responsibility for domestic work remained with the wives, which was attributed to the more traditional views on household division back then [21]. In contrast, our results support the notion that women’s aspirations and role models have changed [8], since women in our sample reported distinct expectations regarding the workers’ engagement in the household.

The fact that conflicts among the couples were described to occur rather seldom could also be related to the coping strategies that were applied, which may be effective in reducing potential conflicts. Moreover, the women in our study seemed to be aware of existing differences compared to non-offshore families; for example, they acknowledged that more compromises had to be made in contrast to other couples. It has been noted previously that such an awareness may increase the implementation of strategies to support family functioning [15].

### 4.3. Coping Strategies

We found the women in our study to apply several coping strategies for dealing with their situation, such as seeking support, thinking positively, and regulating adverse emotions. Thereby, our results undermine previous findings indicating that many offshore women actively pursue some form of coping, e.g., engaging in an active lifestyle or utilizing social support [8,12,22,23]. In earlier studies, women of offshore oil and gas workers were found to use coping strategies to mitigate loneliness when the partner was offshore (e.g., keeping busy, keeping in touch with the family, and taking part in recreational activities [8]). Similarly, FIFO workers were found to engage in social networks providing them with assistance and companionship while the workers were away [12]. Still, there is a need for further investigation of the women’s coping strategies. Aspects such as work schedules (full-time, part-time) and childcare may require different coping strategies that should be examined by further research.

An important strategy of the couples was reported to consist of staying in regular contact, which was also of importance for oil and gas, as well as FIFO, families [8,10,12]. In studies among FIFO families, regular effective communication was the most important strategy to protect family cohesiveness [12], and was found to be strongly associated with family satisfaction [10].

### 4.4. Reconciliation of Offshore Work and Family Life/Partnership

Women’s views regarding the reconciliation of offshore work and family life/partnership seemed to partly depend on their availability of external sources, such as support from friends and family, as well as on the couples’ own expectations. This agrees with the notion that a stronger accordance between perceptions and expectations of partners, e.g., regarding family satisfaction, could lead to less family conflicts [10] and less critical family structures [13].

A support offer provided by offshore companies was named in terms of the flexibility of shifts in cases of emergency. This has also been noted by women of offshore oil and gas workers, who were confident that their partners could be flown home in a family emergency [8]. However, in sum, the women in our study did not report an intensive bandwidth of offers provided by offshore companies to facilitate the reconciliation of offshore work and family life. This finding might either indicate a need for the companies to improve their offers, or to make existing offers more visible for offshore families. In contrast, in FIFO, as well as oil and gas industries, more company support has become evident, e.g., consisting of counseling services, peer-programs, organized family events, or visits to the site for families [8,12]. Such offers may also add to the reconciliation for couples and families in the offshore wind industry.

### 4.5. Strengths and Limitations

A strength of our study is the fact that we recruited women with varying sociodemographic characteristics, e.g., different ages and family status. This enabled us to establish a more complete picture of the situation of offshore women with varying backgrounds. To increase the trustworthiness of our findings, we employed rich descriptions of our results and displayed many direct quotes from the interviewees [25]. Moreover, we discussed our results profoundly within the group of researchers, and contrasted them with empirical references.

However, it should be noted that our findings are based on a convenience sample which was partly achieved via a snowballing technique, thereby increasing the risk of self-selection among the participants. For example, women with a greater interest in the topic might have been more prone to participate, and may not be representative of other female partners of offshore workers. Moreover, our sample is likely to represent a self-selected group of ‘survivors’, as it has been noted for other samples of offshore women [8,21]. There are several indications for this assumption: for example, interviewees’ partners were currently working offshore, and many of them had already worked offshore for several years. It can be assumed that offshore workers are more likely to continue with their work when their women are also able to adjust to the offshore lifestyle [8]. Our sample, therefore, likely represents a survivor group of women that have responded rather positively to this lifestyle and experienced less difficulties in adjustment. Moreover, it should be kept in mind that 10 out of 14 workers were already involved in offshore work at the beginning of the relationship, meaning that most couples did not have to deal with a disrupt change of their living situation during the course of the relationship.

Further methodological limitations concern the fact that we conducted telephone interviews instead of face-to-face interviews, implying an asynchronous communication of place by telephone and a reduction of social clues [26,27].

Another limitation of our study may be seen in the relatively small sample size. However, the size of interviews in our study appeared to be sufficient to achieve data saturation. In support of this, it has been concluded that data saturation usually occurs within the first twelve interviews [28]. In any case, generalizations of our results are impeded by the nature of our qualitative research design.

Further research studies with larger sample sizes are needed. In such studies, it would be interesting to conduct interviews with couples in order to incorporate both the views of offshore workers and their female partners. Moreover, quantitative research studies should be conducted to statistically explore the antecedents, moderators, and outcomes of psychosocial adaptation among offshore couples. Previous research has, for example, suggested that role expectations, the presence of dependent children at home, and the quality of communication in the relationship may influence the effects of stressors on psychosocial adaptation [10]. Moreover, since our sample likely represents a survivor group of couples living the 14/14 schedule, it seems worthwhile to compare their situation with the situation of families where workers decided to leave offshore work.

## 5. Conclusions

The present study expanded upon the current scientific evidence and provided an up-to-date perspective on the situation of offshore wind couples and families living the 14/14 work schedule. The women in our study reported differentiated views as to the benefits and costs associated with their particular lifestyle. Various coping strategies were stated by the women, which could facilitate psychosocial adaptation. Despite experiencing certain burdens, most of the women in our sample seemed to have adapted relatively favorably to their living situation. Our results suggest that certain sociological and technological advances within the last decades, e.g., changes in women’s role models and improved communication technologies, may ease psychosocial adaptation among offshore families.

## Figures and Tables

**Table 1:** Interview topic list.

1	Introduction	Study information, confidentiality, informed consent
2	Socio-demographics	*Interviewee*: age, marital status, occupation, work schedule, duration of partnership with offshore partner, shared household, children *Offshore partner*: offshore experience, occupation, work schedule
3	Phase 1: Single life without offshore partner	Advantages and disadvantages of the phase, contact opportunities while partner is offshore
4	Phase 2: Daily life with offshore partner	Advantages and disadvantages of the phase, associated behavior patterns and feelings
5	Phase 3: Transition phase	Associated behavior patterns and feelings, needs and expectations upon the partner’s arrival
6	Coping strategies	Strategies of the interviewee and the couple
7	Reconciliation of offshore work and family life/partnership	Reconciliation of offshore work with family life/partnership; wishes for a facilitated reconciliation

**Table 2:** Participant characteristics.

**Interviewee**	***n***
**Gender**	
female	14
**Age**	
20–30 years	4
31–40 years	7
41–50 years	3
**Marital status**	
not married	4
married	10
**Work schedule**	
full-time	7
part-time	2
currently in maternity or parental leave	5
**Duration of partnership**	
1–5 years	6
6–10 years	4
11–20 years	2
>20 years	2
**Partnership with partner working offshore from the start**	
yes	4
no	10
**Children**	
yes and living in household	8
expecting	4
no	2
**Offshore partner**	***n***
**Work schedule**	
14 days offshore/14 days onshore	12
other	2
**Offshore experience**	
<1 year	1
1–2 years	6
3–4 years	3
>4 years	4

**Table 3:** Participant characteristics (in detail).

ID	Age *	Marital Status	Occupation	Work Schedule	Duration of Partnership *	Partnership with Partner Offshore *	Shared Household *	Children (in Household)	Occupation	Offshore Experience *	Work Schedule **
**Interviewee**									**Offshore partner**		
1	28	married	not specified (currently on parental leave)	full-time	10	6	9, 5	1 (1)	service technician	6	14/14
2	33	not married	not specified	full-time	3	3	2, 5	0 (0)	technical project management	6	irregular
3	35	married	office management	full-time apprenticeship	10	2, 5	10	2 (2)	quality management	2, 5	14/14 **
4	32	married	social worker (currently on maternity leave)	full-time	5, 5	1, 5	5	0 (0)	health and safety	1, 5	14/14
5	29	married	florist	full-time	2, 5	2, 5	2, 5	1 (1)	service technician	5	8/4
6	39	married	administrative official	part-time	15	5	12	2 (2)	operations manager	5	14/14 **
7	29	married	architect (currently on maternity leave)		10	2	9	0 (0)	offshore medic	2	14/14
8	25	married	maritime sector	full-time	8	0, 5	6	0 (0)	platform master/	0, 5	14/14 **
9	46	married	teacher	full-time	27	3	25	2 (2)	service technician	3	14/14
10	31	married	tailoress (currently on maternity leave)	part-time	5	1, 5	4, 5	0 (0)	nautical officer	1, 5	14/14
11	35	married	health insurance	part-time	13	2	11	1 (1)	service technician	2	14/14
12	34	not married	occupational therapist (currently on maternity leave)	full-time	3, 5	3, 5	0, 5	0 (0)	service technician		14/14
13	42	not married	tailoress	full-time	2, 5	2, 5	1, 5	2 (2)	electrician	3	14/14
14	50	married	house economics	full-time	30	2	28	1 (1)	service technician	2	14/14

* in years ** in turns with office weeks for specific trainings.

**Table 4:** Coping strategies of the women and the couples.

Coping Strategies of the Women	Coping Strategies of the Couples
Seeking support of families and friends	Adhering to fixed contact times
Pursuing an active lifestyle	Mutual understanding and trust
Structuring time in an organized manner	Spending an intensive time together
Adapting oneself to the living situation	
Focusing on time spent together as a couple	
Keeping expectations low	
Contact with other women in the same situation	

## Data Availability

The data analyzed during the current study are not publicly available due to German national data protection regulations. They are available on individual request from the corresponding author.
